# Retraction: Salidroside enhances the anti-cancerous effect of imatinib on human acute monocytic leukemia *via* the induction of autophagy-related apoptosis through AMPK activation

**DOI:** 10.1039/d4ra90050b

**Published:** 2024-04-29

**Authors:** Chiyu Ge, Junli Zhang, Feng Feng

**Affiliations:** a School of Pharmacy, Jiangsu Food and Pharmaceutical Science College Meicheng Road No. 4 Huaian City Jiangsu Province P. R. China zhangjunlijl@sina.com

## Abstract

Retraction of ‘Salidroside enhances the anti-cancerous effect of imatinib on human acute monocytic leukemia *via* the induction of autophagy-related apoptosis through AMPK activation’ by Chiyu Ge *et al.*, *RSC Adv.*, 2019, **9**, 25022–25033, https://doi.org/10.1039/C9RA01683J.

The Royal Society of Chemistry hereby wholly retracts this *RSC Advances* article due to concerns with the reliability of the data in the published article.

Many panels in Fig. 2b have been duplicated in other publications by different authors (T. Liu, A. Li, Y. Xu and Y. Xin, *Cancer Med.*, 2019, **8**, 751–760; N. Tang, Y. Dong, C. Chen and H. Zhao, *Front Pharmacol.*, 2020, **11**, 519172; and Y. Zhang, H. Du, X. Yu and J. Zhu, *Ann Transl Med.*, 2020, **8**, 1501).

The ‘Ctrl/Kidney’ panel in Fig. 6c is duplicated in another publication by different authors (Y. Tian, J. Shu, R. Huang, X. Chu and X. Mei, *Transl Androl Urol.*, 2020, **9**, 1356–1365).

The ‘Liver/Salidroside + Imatinib’ panel in Fig. 6c is duplicated in another publication by different authors (W. Yan, K. Li, A. Buhe, T. Li, P. Tian and J. Hong, *RSC Adv.*, 2019, **9**, 25655–25666).

The authors were asked to provide the raw data for this article, but did not respond. Given the significance of the concerns about the validity of the data, and the lack of raw data, the findings in this paper are not reliable.

The authors have been informed but have not responded to any correspondence regarding the retraction.

Signed: Laura Fisher, Executive Editor, *RSC Advances*

Date: 17th April 2024

## Supplementary Material

